# Identification of determinants of high-fidelity DNA synthesis in *Mycobacterium smegmatis* DnaE1 through *in silico* and *in vivo* approaches

**DOI:** 10.1093/nar/gkaf1274

**Published:** 2025-11-24

**Authors:** Rosan C M Kuin, Gerard J P van Westen, Meindert H Lamers

**Affiliations:** Medicinal chemistry, Leiden University,Einsteinweg 55, 2333 CC Leiden, the Netherlands; Department of Cell and Chemical Biology, Leiden University Medical Center,Albinusdreef 2, 2333 ZA Leiden, the Netherlands; Medicinal chemistry, Leiden University,Einsteinweg 55, 2333 CC Leiden, the Netherlands; Department of Cell and Chemical Biology, Leiden University Medical Center,Albinusdreef 2, 2333 ZA Leiden, the Netherlands

## Abstract

Drug resistance in *Mycobacterium tuberculosis* presents a major challenge in tuberculosis treatment, highlighting the need to understand the underlying mechanisms. DNA replication plays an important role in the acquisition of drug resistance, and the expression of the DNA polymerase DnaE2 during adverse conditions has been associated with increased mutation rates. Here, we investigate the functional differences between the high-fidelity replicative DNA polymerase DnaE1 and the predicted error-prone DNA polymerase DnaE2, focusing on which amino acid changes affect polymerase fidelity. For this, we identify potential fidelity-altering positions using a two-entropies sequence analysis combined with experimental validation to test whether changes of these positions affect the mutation rates. We find that a double mutation in the palm domain of *Mycobacterium smegmatis* DnaE1: D431S/R432D, increases mutation frequencies both *in vivo* and *in vitro*. The location of these two residues adjacent to the DNA backbone of the template strand suggests that the amino acid change results in a looser grip on the DNA, allowing for the incorporation of incorrect nucleotides. These insights improve our understanding of the mechanisms underlying drug resistance in *M. tuberculosis* and could help in the development of future strategies to combat it.

## Graphical Abstract

**Graphical Abstract:**
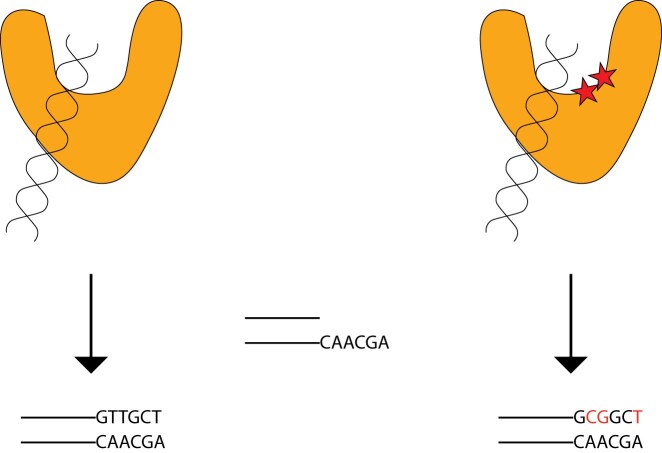

## Introduction

*Mycobacterium tuberculosis*, the pathogen responsible for tuberculosis, remains the top infectious killer globally [1, 2]. The emergence of resistant forms of tuberculosis and in particular multidrug- and extensively drug-resistant tuberculosis form a growing threat to global health [3, 4]. These resistant forms of tuberculosis require lengthy treatment regimens with significant side effects and consume a substantial amount of the healthcare budget and associated resources in endemic countries [5, 6]. Hence, a better understanding of the mechanisms behind drug resistance is needed to be able to develop therapeutic interventions to slow down drug resistance.

Unlike many other bacteria, resistance in *M. tuberculosis* is not acquired via horizontal gene transfer [7, 8]. Instead, the driver of drug resistance in *M. tuberculosis* is the acquisition of mutations during DNA replication in genes that encode drug targets or drug-activating enzymes [7, 9]. Therefore, targeting DNA replication and more specifically the bacterial DNA polymerase would be an attractive approach for slowing down drug resistance [10, 11].

Mycobacteria contain two copies of a C-family DNA polymerase: the high-fidelity replicative DNA polymerase DnaE1 and DNA polymerase DnaE2, which is expressed under adverse conditions, such as DNA damage or antibiotic exposure [8, 12–14]. While DnaE1 ensures accurate DNA replication, the expression of DnaE2 has been associated with increased mutation rates, reflecting its role in adaptive responses [13, 15]. Unlike DnaE1, which is functionally organized into the replisome, DnaE2 interacts with other proteins—ImuA' and ImuB—to form the mutasome, a complex crucial for its error-prone activity [16].

Like other C-family DNA polymerases, DnaE2 contains both a polymerase active site and an exonuclease active site [17, 18]. The importance of the polymerase active site is highlighted by the observation that changes in the polymerase active site of DnaE2 reduce mutation rates in *M. tuberculosis* [14]. In contrast, the exonuclease domain of DnaE2 is predicted to be inactive due to the absence of a critical metal-binding residue [16]. The predicted lack of a functional exonuclease domain in DnaE2 fits well with its association with higher mutation rates, as inactivating mutations in the exonuclease domain of DnaE1 lead to increased mutation rates in the bacterium [16, 19, 20]. However, whether DnaE2 also incorporates nucleotides with reduced accuracy remains an open question.

So far, no biochemical studies involving DnaE2 have been published, possibly due to the difficulty of working with DnaE2 in isolation [15]. To work around these challenges, we employed a two-entropies analysis of 358 DnaE1 and DnaE2 sequences to identify amino acid positions that potentially influence fidelity of DNA synthesis. Next, we created 16 DnaE1 variants by replacing these amino acids with their corresponding residues from DnaE2 and tested these in *Mycobacterium smegmatis* through dCas9-mediated knockdown of endogenous DnaE1 and rescue by the variant DnaE1. Variants with increased mutation rates were subsequently purified and analysed *in vitro* for fidelity of DNA synthesis.

Our results identify a double mutant in the palm domain of *M. smegmatis* DnaE1: D431S/R432D, which exhibits enhanced mutation frequencies in both phenotypic and biochemical settings. The location of the double mutation adjacent to the backbone of the template strand suggests that the alteration may create a more open active site that is more generous to mis-incorporated nucleotides. We also confirmed the predicted lack of exonuclease activity in DnaE2 by changing key residues in the PHP domain that is responsible for exonuclease activity in DnaE1. Combined, these results indicate that DnaE2 acts as an error-prone polymerase that introduces more errors during catalysis, providing novel insights into the functioning of the *M. smegmatis* mutasome.

## Materials and methods

### Materials

All chemicals were purchased from Sigma–Aldrich unless stated otherwise. DNA substrates were ordered from IDT.

### Two-entropies analysis of DnaE1 and DnaE2 sequences

Homologous DnaE1 and DnaE2 sequences were obtained using BLAST [21]. To mitigate bias, we included one sequence per organism. The taxonomy was restricted to only include Mycobacteria that are known to contain DnaE1 and DnaE2 genes [17, 22]. The resulting 358 sequences (179 per family) were aligned using ClustalOmega [23]. Subsequently, we used the Shannon entropy to measure sequence conservation at each amino acid position in the sequence alignment, as given by Formula 1 below. Here, the Shannon entropy (SE) at position *i* in the multiple sequence alignment is given by *a* that loops over the 20 different amino acids, and *f_ia_* is the fraction of residues of type *a* at alignment position *i*. Each gap was treated as a different residue so that the conservation for highly gapped positions is low.


\begin{eqnarray*}
S{{E}_i} = - \mathop \sum \limits_{a = 1}^{20} {{f}_{ia}} \cdot {{\log }_2}{{f}_{ia}}
\end{eqnarray*}

The Shannon entropy calculations were done for all DnaE1 and DnaE2 sequences combined, as well as for DnaE1-only and DnaE2-only sequences separately, using a two-entropies analysis as described [24, 25]. The entropy values for DnaE1 and DnaE2 were summed, and values were scaled between 0 and 1. By adding the separate entropy values of DnaE1 and DnaE2, differentially conserved residues can be identified by focusing on residues that have a low protein-specific entropy value and a higher overall entropy value.

### Selection of final set of residues for experimental validation

For making a final selection of differentially conserved residues for experimental validation, we focused on residues with different physicochemical properties between corresponding residues in DnaE1 and DnaE2. For this, we use different physicochemical descriptor variables, called Z-scales [26]. To measure the variability of Z-scales in the multiple sequence alignment, we calculated the standard deviation per position and scaled these values between 0 and 1.

In addition, to exclude mutations that are predicted to have a negative impact on protein stability, we estimated the changes in free energy upon mutation for all possible DnaE1 to DnaE2 mutations using ICM-Pro version 3.9-3a (Molsoft L.L.C.) [27]. The free energy change (ΔΔG) in protein stability was then calculated as the difference between the Gibbs energy (ΔG) of the mutant and the wild type. In a similar way, the predicted change in binding free energy (ΔΔGbind) to the DNA was calculated upon mutation. The final selection was made by visual inspection and selecting residues close to the DNA, as these were expected to have the biggest effect on fidelity.

### Preparation of dCas9 knockdown plasmids

Knockdown of *M. smegmatis* DnaE1 was achieved by using a catalytically dead Cas9 (dCas9). sgRNA oligos were designed using the Pebble design workflow [28]. The primer pair was annealed and cloned into the anhydrotetracycline (ATc)-inducible vector plJR962 (Addgene, #115162) using restriction cloning as described [28, 29]. Briefly, plJR962 was digested with BsmBI-v2 and gel purified. Two complementary oligonucleotides were then annealed and cloned into the digested plasmid backbone. Different oligo pairs were tested, and the one predicted to be the strongest repressor was selected for DnaE1 (top: 5′-GGGAAGACCGTGGTCCATCAGTTCGAG-3′, bottom: 5′-AAACCTCGAACTGATGGACCACGGTCT-3′), targeting the PAM sequence corresponding to residues 196–198 of DnaE1. As a control for increased mutagenesis, we targeted the EndoMS/NucS enzyme that is responsible for the post-replicative removal of mismatches in mycobacteria [30]. A NucS-targeting primer pair (top: 5′- GGGAGGCGAACACACCTGCCACCGGCG-3′, bottom: 5′- AAACCGCCGGTGGCAGGTGTGTTCGCC-3′) was obtained and cloned as described above. Plasmid construction was performed in *Escherichia coli* DH5α cells, grown in Luria–Bertani medium at 37°C. Antibiotics were used at the following concentrations: kanamycin at 50 µg/ml, chloramphenicol at 35 µg/ml, and hygromycin at 200 µg/ml.

### Preparation of CRISPRi-resistant DnaE1 variant plasmids

The *M. smegmatis* DnaE1 gene (UniProt ID: A0QX55) was cloned in the pINIT vector (Addgene, #46858) using FX-compatible primers and the FX protocol [31]. Two silent mutations in codons 196 (AAC → AAT) and 198 (TTC → TTT) were introduced in the sgRNA target sequence of the DnaE1 expression plasmid to make it CRISPRi resistant without changing the amino acid sequence. DnaE1 mutants were generated by using commercial dsDNA gene fragments (IDT) and cloning them in the sgRNA-resistant pINIT vector using In Vivo Assembly [32]. The gene fragments and associated primers were designed using the Python package PyVADesign [33]. Mutants were then subcloned in a modified pACE expression vector without a GFP tag for acetamide-inducible expression using FX cloning [31]. Plasmid construction was performed in *E. coli* DH5α cells, as described above.

### Preparation of *M. smegmatis* cells expressing dCas9 and DnaE1 variants

*Mycobacterium smegmatis* MC^2^155 were transformed with the dCas9 plasmid and DnaE1 plasmid in two steps. Cells were made competent as described [34], transformed with 1 µg dCas9 plasmid by electroporation, and plated on 7H10 agar plates. Single colonies were selected and made competent a second time and subsequently transformed with the DnaE1 variant plasmid. The resultant colonies were used for whole-cell analysis of DnaE1 variants. *Mycobacterium smegmatis* MC^2^155 was grown aerobically at 37°C in Middlebrook 7H9 medium containing 0.2% (v/v) glycerol and supplemented with 10% (v/v) albumin–dextrose–catalase (ADC) growth supplement and 0.005% (v/v) Tween-80. Solid media consisted of 7H10 agar containing 0.2% (v/v) glycerol and 10% (v/v) ADC. Antibiotics were used at the following concentrations: kanamycin at 50 µg/ml, hygromycin at 100 µg/ml, and rifampicin at 200 µg/ml.

### Whole-cell analysis of DnaE1 mutants

*Mycobacterium smegmatis* MC^2^155 cells transformed with dCas9 and a DnaE1 variant were analysed for both growth defects and changes in mutagenesis rates. To measure the effect of the DnaE1 variant on bacterial growth, cells were recovered for 3 h after transformation at 37°C while shaking at 200 RPM, and the culture was split into four parts. Each part was plated on solid media containing 0.4% (w/v) acetamide for DnaE1 expression and/or 100 ng/ml anhydrotetracycline for dCas9 expression. Plates were imaged four days after transformation, and the experiment was repeated independently three times.

To measure the effect of the DnaE1 variants on mutagenesis, we counted the number of rifampicin-resistant colonies as an indirect measure of the mutagenesis rate, following the method previously reported [13, 35]. For this, cells expressing dCas9 and a DnaE1 variant were grown in a liquid culture to an OD_600_ of 0.4–0.5. Next, 5 ml was plated onto 7H10 solid media containing 200 μg/ml rifampicin. As a control, a dilution series was spotted on solid media without rifampicin to estimate the colony-forming unit (CFU). Colonies were counted after four days, and mutation frequencies were calculated by dividing the number of rifampicin-resistant colonies by the average CFU/ml of the dilution series. Each experiment was performed in triplicate and repeated in three independent measurements.

### Protein purification

*Mycobacterium smegmatis* DnaE1 WT and mutants, containing an N-terminal His-tag, were expressed in *M. smegmatis* MC^2^155 cells. Cells were grown in 1× YT media at 37°C while shaking at 200 RPM, and protein production was induced at OD_600_ ∼0.7 with 0.4% (w/v) acetamide for 6 h at 30°C. Cells were lysed by sonication, and proteins were purified using a Histrap column (Cytiva) using 25–500 mM imidazole, 50 mM HEPES (pH 7.5), 500 mM NaCl, and 1 mM DTT, followed by a heparin column (Cytiva) using 50 mM HEPES (pH 7.5), 0.1–1.0 M NaCl, and 1 mM DTT. The obtained proteins were found to be at a concentration of 20–30 μM, flash-frozen in liquid nitrogen, and stored at −70°C until use.

### Gel-based DNA polymerase assays

The error-proneness of DnaE1 and its mutants was assessed under two conditions: a manganese-dependent assay and a time-course experiment. Both assays were performed in 50 mM HEPES (pH 7.5), 50 mM potassium glutamate, 6 mg/ml BSA, 2 mM DTT, and 5 mM MgCl_2_. Reactions were performed at 20°C and in the presence of 100 μM dNTPs (each).

For the manganese-dependent assay, 50 nM of purified protein and 50 nM DNA substrate (for DNA sequences see Supplementary Table S1) were used. Primer extensions were done for 30 min with increasing MnCl_2_ concentrations from 0.3 to 5 mM. Reactions were stopped in 50 mM EDTA (pH 7.4), separated on a 14% native acrylamide gel, stained with SYBR Safe, and imaged with a Bio-Rad Gel Doc XR+ system. For the time-course experiment, 100 nM of purified protein and 100 nM of FAM-labelled DNA substrate were used (see Supplementary Table S1). Primer extensions were done for 80 min (time points taken at 5, 10, 15, 20, 30, 40, and 80 min), and reactions were stopped in 20 mM EDTA (pH 7.4) and 75% formamide and analysed on a denaturing 20% acrylamide/bis-acrylamide (19:1) gel with 7.5 M urea in 1× TBE for 40 min at 30 W. The gel was imaged with a Typhoon Imager (GE Healthcare).

For both assays, three independent experiments were performed for quantification of extended product formation using ImageLab software 6.0.1 (Bio-Rad). In the manganese-dependent assay, the fraction of fully extended product was calculated as a proportion of the total signal per lane, whereas in the time-course experiment, the fraction of fully extended product was normalized to the maximum signal intensity observed for the fully extended product.

## Results

### Design of DnaE1 variants for altered fidelity based on two-entropies analysis

**Figure 1.**
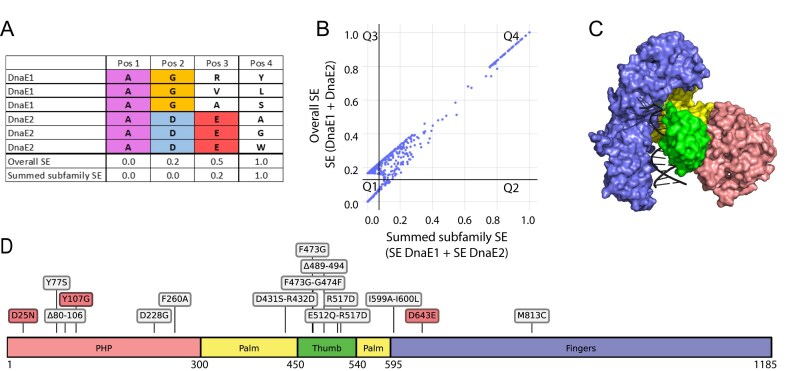
Two-entropies analysis and selection of DnaE1 variants for experimental validation. (**A**) Example positions of a Multiple Sequence Alignment for illustrating the types of positions that were identified in the two-entropies analysis. Residues conserved in both families receive a low score in overall SE and in the summed subfamily SE (Pos 1). Residues differently conserved per family receive a higher overall SE but a low score for the summed subfamily SE (Pos 2). Residues conserved in only one family are marked by a higher overall SE and slightly elevated summed subfamily SE (Pos 3). Finally, residues that are not conserved in either family are marked by a high overall SE and high summed subfamily SE (Pos 4). (**B**) 2D plot with summed subfamily SE shown on the horizontal axis and overall SE shown on the vertical axis (scaled between 0 and 1). Each dot represents a position in the multiple sequence alignment of 358 homologous DnaE1 and DnaE2 sequences from mycobacteria. Two lines (x = 0.07 and y = 0.13) mark the separation of the residues into four quadrants, based on visual inspection. Example positions Pos 1–4 from panel A would approximately correspond to Q1, Q3, Q3, and Q4, respectively. (**C**) Domains (Palm, Fingers, Thumb, PHP) of DnaE1 mapped onto the structure (PDB ID: 7PU7). (**D**) Distribution of the 16 selected DnaE1 variants across the protein sequence, based on the two-entropies analysis. Control variants for increased mutagenesis are shown in red, and variants selected based on the two-entropies analysis are shown in grey.

To identify positions that could affect the fidelity of DnaE1, we used a multiple sequence alignment of 358 DnaE1 and DnaE2 sequences. To identify positions that are conserved within each polymerase family but are divergent between them, a two-entropies analysis was applied using the SE as a measure for sequence conservation [24, 25]. SE calculations were performed for DnaE1-only and DnaE2-only sequences, as well as for all sequences combined (Fig. 1A). By summing up the separate SE values of DnaE1 and DnaE2 (SE DnaE1 + SE DnaE2) and comparing these to the overall Shannon entropy values SE (DnaE1 + DnaE2), differentially conserved residues can be identified by focusing on residues that have a low protein-family-specific entropy value and a high overall entropy value. These differentially conserved residues may be crucial for maintaining the specific enzymatic functions of each polymerase, such as polymerase fidelity. The results of the two-entropies analysis, presented in Fig. 1B, show a linear plot, which can be attributed to the limited variability between DnaE1 and DnaE2 sequences, which share an average sequence identity of 55%. To make the two-entropies analysis plot easier to interpret, it has been separated into quadrants with the lines crossing at the point where we can discriminate between overall-conserved and subfamily-conserved residues.

**Figure 2.**
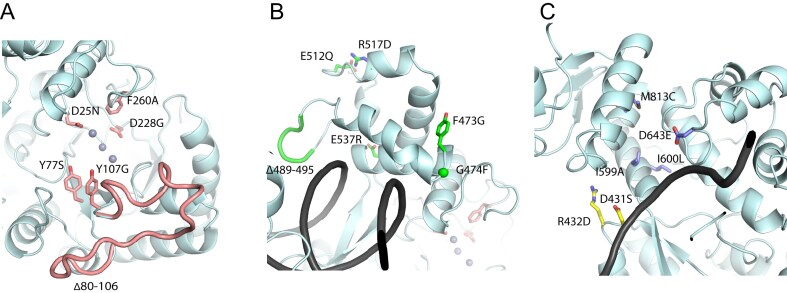
Mutated residues in DnaE1. (**A**) Residues in the PHP domain, shown as pink sticks. (**B**) Residues in the thumb domain, shown as green sticks. (**C**) Residues in the palm and fingers domain, shown as yellow and purple sticks, respectively.

Residues in quadrant 1 (Q1, lower left corner) are globally conserved between both polymerase families, such as the *M. smegmatis* polymerase active site residues 424, 426, and 590 that are essential for catalysis in both DnaE1 and DnaE2 [14, 17]. In contrast, residues in Q4 (upper right corner) are those that are not conserved in either of the two families. Curiously, Q4 also contains residues located in loops that are present in the DnaE1 family but not in DnaE2-family polymerases. One of these loops (residues 80–106) is located in the PHP domain (Figs 2 and 1C and D for domain definition). The other loop (residues 489–495) is located in the thumb domain and inserts itself into the major groove of the DNA [12] (Fig. 2). To investigate whether these loops affect polymerase fidelity, two DnaE1 variants were designed, each with one of the loops removed.

Quadrant 3 (Q3) shows residues that are conserved in either DnaE1 family or DnaE2 family, but not in both, and may therefore play a role in the distinct enzymatic behaviour of the two proteins. It is important to note that differences between DnaE1 and DnaE2 are not only limited to the fidelity of DNA synthesis but could also include residues that are required to interact with partner proteins of the replisome or mutasome. Therefore, to maximize the chances of selecting alterations that affect fidelity, we focused on a subset of residues near the DNA-binding region. To further enhance the likelihood that alterations impact DNA synthesis, we focused on residues with different physicochemical properties between corresponding residues (Supplementary Fig. S1). Finally, to lower the chances of selecting mutations that could destabilize the protein, the predicted effect on protein stability upon mutation was calculated using the program ICM-Pro (see the “Materials and methods” section). Finally, double mutations were designed when two differentially conserved residues were in close proximity to each other in the protein structure to take into account their potential compensatory effect. Based on these criteria, a total of 11 positions were selected for experimental validation (Fig. 1D and Supplementary Fig. S1).

To evaluate the effect of the selected positions on polymerase fidelity, variants of DnaE1 were created in *M. smegmatis* by replacing the DnaE1 residue with the corresponding DnaE2 residue (Supplementary Fig. S2). The complete selection of DnaE1 variants contains 11 amino acid variants and two loop deletions based on the two-entropies analysis, and three control mutations based on previously reported effects on DnaE1 (Table 1 and Fig. 2). Two of the control mutations, D25N and Y107G, are in the PHP domain and were selected based on their inability to remove mis-incorporated nucleotides from a DNA substrate [36]. A third control mutation in the fingers domain, D643E, was selected due to the mutator phenotype associated with this mutation in the corresponding residue of *E. coli* Pol IIIα [37]. In addition, as positive controls for increased mutagenesis, we included a plasmid expressing *M. smegmatis* DnaE2, as well as a dCas9 knockdown of NucS, the key mycobacterial mismatch repair enzyme [30].

**Table 1.** Variants of *M. smegmatis* DnaE1 used in this study

			Cell growth		Mutation frequency	
	Mtb residue	Domain	- WT DnaE1	+ WT DnaE1	- WT DnaE1	+ WT DnaE1
DnaE1 WT (*control*)			=	=	n.d.	1.0
DnaE2 (*control*)			∅	=	n.d.	2.8 ± 0.7
ΔNucS (*control*)			=	=	n.d.	64.5 ± 23.7
D25N (*control*)	23	PHP	∅	<	n.d.	3.4 ± 2.1
Y107G (*control*)	105	PHP	=	=	22.1 ± 5.6	n.d.
F260A	258	PHP	∅	<	n.d.	5.9 ± 2.7
F473G	470	Thumb	∅	<	n.d.	1.9 ± 0.9
F473G-G474F	470–471	Thumb	∅	<	n.d.	1.2 ± 0.9
Δ489–495	486–492	Thumb	∅	<	n.d.	n.d.
E512Q-R517D	509–514	Thumb	∅	<	n.d.	0.2 ± 0.1
R517D	514	Thumb	∅	<	n.d.	1.1 ± 0.3
E537R	534	Thumb	∅	=	n.d.	n.d.
D431S-R432D	428–429	Palm	=	=	7.0 ± 1.9	n.d.
I599A-I600L	596–597	Fingers	<	=	0.5 ± 0.1	n.d.
D643E *(control)*	640	Fingers	<	=	0.9 ± 0.8	n.d.
M813C	810	Fingers	=	=	0.9 ± 0.5	n.d.

Effect on cell growth upon knockdown of endogenous DnaE1 and rescue by variants, measured across three biological replicates. ∅ indicates that there was no growth, < indicates that there was less growth compared to the WT, and = indicates that there was equal growth as compared to the WT, four days after transformation. Effect of the DnaE1 variant on mutation frequencies, determined using a rifampicin resistance assay, reported as fold increase (n.d. = not determined) ± the standard deviation across three biological replicates.

### Phenotypic screen using dCas9-mediated knockdown of DnaE1 reveals little tolerance for variation in DnaE1

The DnaE1 variants selected above were tested in *M. smegmatis* through dCas9-mediated knockdown of endogenous DnaE1 and rescue by the variant DnaE1. For ease of use, we used the non-pathogenic, fast-dividing *M. smegmatis* that shows high genetic resemblance to *Mycobacterium tuberculosis* (Supplementary Fig. S3 and S4) and conservation of DNA replication and mutagenesis mechanisms [38, 39]. In this setup, knockdown of DnaE1 results in no growth, consistent with the essential role of DnaE1 [40], which can be rescued by plasmid-based expression of dCas9-resistant DnaE1 (Supplementary Fig. S5). Next, we evaluated the effect of the DnaE1 variants on cell growth by growing them in the presence and absence of the genomic DnaE1 (Table 1 and Supplementary Table S2). Surprisingly, the majority of DnaE1 variants could not grow in the absence of the genomic DnaE1, indicating that DnaE1 is intolerant to mutations across all domains (PHP, thumb, palm, fingers; see Fig. 1C and D). In addition, most DnaE1 variants also showed reduced growth when genomic DnaE1 was present, indicating a dominant negative effect. This suggests that the presence of the mutant polymerase disrupts the DNA synthesis by competing with the wild-type DnaE1 for a place in the replisome. We also tested whether DnaE2 could replace DnaE1, which resulted in no growth. In contrast, expression of DnaE2 in the presence of WT DnaE1 did not show any impact on growth, unlike many of the DnaE1 variants. This not only suggests that DnaE2 cannot replace DnaE1, but that it also cannot interact with the replisome, as it does not exert a dominant negative effect. Instead, DnaE2 has evolved to interact with the mutasome [14].

### The double mutant D431S/R432D shows increased mutation frequencies

To measure the effect of the DnaE1 variants on fidelity of DNA synthesis, we used the appearance of rifampicin-resistant colonies as a measure for increased mutagenesis [13, 41]. The setup of this assay was confirmed by measuring the mutation frequency of a dCas9 knockdown of NucS, the key enzyme of the non-canonical DNA mismatch repair pathway in mycobacteria [30]. Knockdown of NucS results in a ∼64-fold increase in mutation frequency (Table 1). Also, the expression of DnaE2 in the presence of the genomic DnaE1 increases mutagenesis, but only by three-fold. The limited impact on mutagenesis upon expression of DnaE2 may be explained by the lack of the other mutasome components, ImuA’ and ImuB, as it was reported that deletion of any of the mutasome components eliminated mutagenesis in *M. smegmatis* [14, 42].

Next, we tested the effect of the DnaE1 variants on mutagenesis. In the PHP domain, only one of the variants could be expressed in the absence of the wild-type gene. This control variant, Y107G, shows a 22-fold increase in the number of rifampicin-resistant colonies. This is consistent with previous work that showed that this mutant lacks exonuclease activity [36]. As the remaining variants in the PHP domain could not rescue the wild-type gene, we measured their impact on mutagenesis in the presence of the endogenous DnaE1 (Table 1 and Supplementary Table S2). We find that co-expression of the D25N and F260A variants with wild-type DnaE1 led to a 3.4-fold and 5.9-fold increase in mutagenesis (Table 1), respectively. The smaller impact on mutagenesis compared to Y107G may be explained by the presence of the wild-type DnaE1 that attenuates their impact on mutagenesis. Other PHP variants (Y77S, D228G, and Δ80–106) did not increase mutagenesis (Supplementary Table S2). The lack of effect for D228G may be due to protein instability [36], and for Y77S and Δ80–106, it could be either that the mutation does not affect exonuclease activity or that they result in unstable protein too. Thumb domain variants, expressed only with wild-type DnaE1, negatively affected bacterial growth but did not increase mutagenesis, suggesting that their mutation effects or compromised polymerase activity, leading to e.g. stalled DNA synthesis. Variants in the fingers domain, expressed without the wild-type protein, showed no impact on mutagenesis, indicating no effect on polymerase fidelity. In contrast, the variant in the palm domain: D431S/R432D, showed a seven-fold increase in mutagenesis without having an impact on bacterial growth.

**Figure 3.**
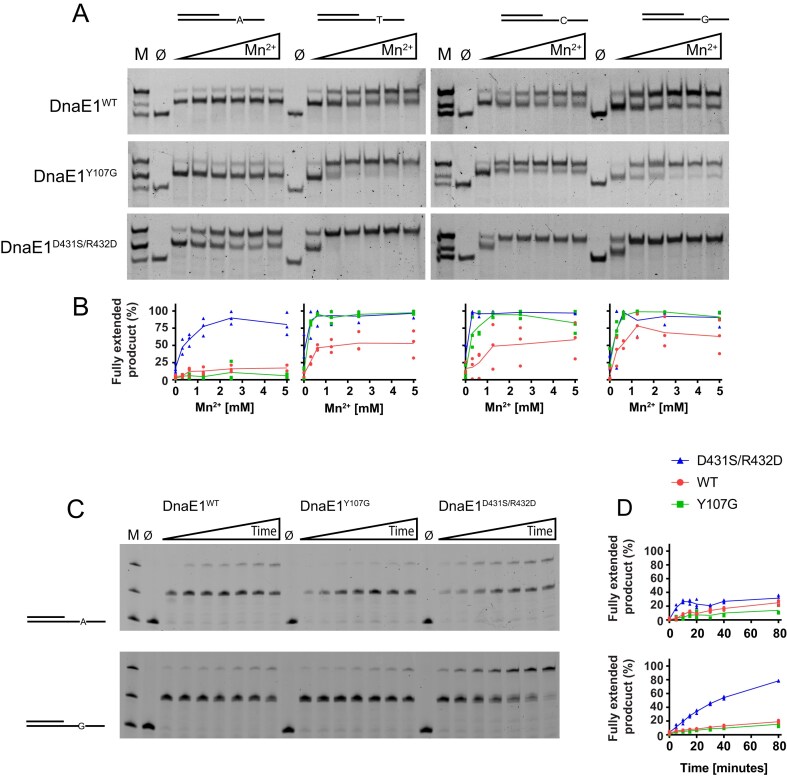
Polymerase assay of wild-type and variant *M. smegmatis* DnaE1. (**A**) Four different substrates were used, each with a unique nucleotide in the single-stranded region of the template DNA. Extension reactions were performed with only three of the four nucleotides, thus requiring the polymerase to create a mismatch for complete extension of the primer strand. Increasing amounts of manganese (Mn^2+^: from 0.3 to 5 mM) were added to enhance the error rate of the polymerase. Reaction products were resolved on a native acrylamide gel and stained with SYBR Safe. Marker lane (M) shows the start substrate (bottom band), the partially extended substrate (middle band), and the fully extended substrate (top band). ∅ marks the lane in which all nucleotides were omitted from the reaction. (**B**) Quantification of three independent experiments (see Supplementary Fig. S6), showing the percentage of fully extended product relative to the total signal in each lane. Individual data points and mean values are plotted. (**C**) Time-course assay with DNA substrates A and G without addition of Mn^2+^. Reaction products were resolved on a denaturing urea-acrylamide gel and visualized through the 5′ fluorescently labelled primer strand. Timepoints were taken at 5, 10, 15, 20, 30, 40, and 80 min. (**D**) Quantification of three independent experiments (see Supplementary Fig. S7), showing the percentage of fully extended product, normalized to the maximum signal intensity observed.

Hence, from the 16 variants tested, only two variants, Y107G and D431S/R432D, showed increased mutagenesis without impacting the growth of the bacterium. For further analysis, these two variants were purified and their activity was assessed *in vitro*. To do so, we monitored DNA polymerase activity using four specially designed DNA substrates. In each of these, the template strand consists of an 18-nucleotide, single-stranded 3′ overhang containing only three of the four nucleotides with a single fourth nucleotide placed at the centre (Fig. 3A). Next, by providing only three of the complementary nucleotides, the polymerase will stall on the single nucleotide, unless it is able to insert and extend from a mismatch. To enhance the number of mismatches, we added increasing amounts of manganese, which is known to promote the error rate of the polymerase [43], thus increasing the likelihood that a mismatch is made.

For the wild-type protein, we find that in the absence of manganese, the polymerase rarely makes it past the middle of the single-stranded overhang where the fourth nucleotide is positioned. Addition of manganese increases the amount of fully extended product, although much of the stalled product remains. In the Y107G variant that lacks exonuclease activity [36], we find that the amount of fully extended product is increased for substrates with the T, C, and G compared to the wild-type protein (Fig. 3A and B and Supplementary Fig. S6). The amount of fully extended product is almost complete in the D431S/R432D variant, indicating that this double mutation in the palm domain strongly enhances the propensity of the polymerase to incorporate and extend a mismatch. Curiously, for the substrate with the single A in the overhang, the addition of manganese shows no effect for the wild-type protein or the Y107G variant, whereas the palm mutant D431S-R432D shows an increase of fully extended product upon the addition of manganese.

To further analyse the differences between the wild-type and variant DnaE1 polymerases, we measured their extension rates over time, without the addition of manganese (Fig. 3C-D and Supplementary Fig. S7). For this we used a 5′ fluorescently labelled primer strand and high-resolution denaturing gel electrophoresis to resolve single-nucleotide extension of the DNA substrates. We furthermore only used the A and G substrates, as the C and T substrates behaved identical to the G substrate in the manganese-dependent assay. In the time-dependent assay, we find that on the A-substrate all three DnaE1 proteins rarely make it past the single A in the template strand, with the amount of fully extended product after 80 min ranging from 14% for the Y107G variant to 25% for wild-type protein and 32% for the D431S/R432D variant. For the G-substrate, the Y107G variant and wild-type protein produce similar amounts of full-length product (15% and 19%, respectively), while the amount of full-length product produced by the D431S/R432D variant has increased 2.4-fold to 78% when compared to the A-substrate. For all three proteins, we observe only a single band at the position of single A or G in the template strand. This band coincides with the size of the pre-insertion product, i.e. before the wrong nucleotide is inserted into the primer strand. This suggests that the strongest contributor to the fidelity of DnaE1 occurs at the insertion step, which is supported by the exonuclease-deficient Y107G variant that shows no additional +1 insertion products. Therefore, the increased propensity of the D431S/R432D variant to synthesize past the single nucleotide in the template strand is likely caused by a lowering of the insertion fidelity that allows for a more frequent insertion of a wrong nucleotide, from which extension to the full-length product can take place. The lack of increased synthesis past the single A by the D431S/R432D cannot be readily explained but suggests that any of the possible mismatches opposite the A is such a poor fit for the polymerase active site, that even the reduced fidelity variant cannot easily make it.

### The location of the D431S/R432D suggests a more open DNA binding groove

**Figure 4.**
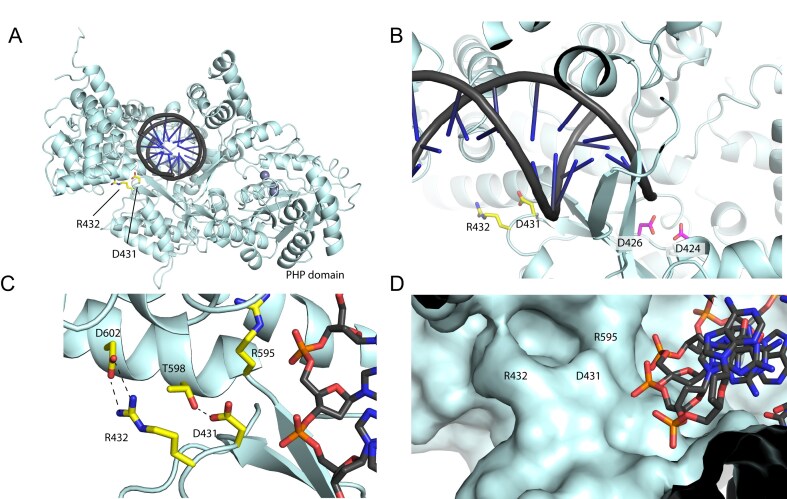
Analysis of DnaE1 D431S/R432D. (**A**) Location of the D431 and R432 in the Palm domain of DnaE1. (**B**) Close up of the position of the two residues adjacent to the template strand and immediately downstream of the catalytic residues D424 and D426 (**C**) Hydrogen bonding between D431 and R432 to two residues in the Fingers domain: T598 and D602. (**D**) Surface representation of the Palm domain, showing a groove for the DNA template strand. Location of D431, R432, and R595 is shown.

As shown above, mutations in the PHP domain result in an increased mutagenesis, which is consistent with various observations that inactivation of the proofreading activity increases mutation rates [16, 36, 44]. The location of the mutation-inducing variant D431S/R432D, on the other hand, is >40 Å away from the variants in the PHP domain (Fig. 4A), suggesting it acts in another way. The two residues are located adjacent to the sugar-phosphate backbone of the template strand, two base pairs away from the insertion site of the incoming nucleotide (Fig. 4B). They are located immediately after the strand that holds two of the catalytic residues of the polymerase active site: D424 and D426 (Fig. 4B). Interestingly, D431 and R432 are close to, but do not contact, the DNA backbone. Instead, they form hydrogen bonds with two residues in the fingers domain: T598 and D602 (Fig. 4C). Doing so, the hydrogen bonds stabilize the position of D431 and R432 so that together with R595 they create a groove that follows the DNA backbone (Fig. 4D). In an AlphaFold [45] model of the D431S/R432D variant, the interaction with T598 and D602 is lost, causing the groove to widen and appear “open” (Supplementary Fig. S8). The increased mutation rate observed for the D431S/R432D variant could be the result of this wider DNA-binding groove that enables the DNA to adapt to wrongly incorporated nucleotides. Alternatively, or in addition, the lack of hydrogen bonds in the D431S/R432D variant may allow for increased movement of the catalytic residues D424 and D426, located immediately upstream of the double mutation, that thereby become more generous to mis-incorporated nucleotides. A comparison with an AlphaFold [45] model of DnaE2 supports this interpretation. In DnaE2, the residues corresponding to D431 and R432 (S465 and D466), only D466 appears to be able to form hydrogen bonds with Y610 of the fingers domain (Supplementary Fig. S8). Moreover, no equivalent groove is observed, suggesting that the DNA may adopt a different orientation relative to the catalytic residues in DnaE2. It should be noted, though, that the reliability of the DnaE2 model, particularly with respect to sidechain conformations, may be limited since no experimentally determined structures of closely related proteins are available.

## Discussion

Tuberculosis poses a significant global health risk, a situation worsened by the frequent emergence of drug-resistant strains. Resistance is often associated with *de novo* point mutations in the DNA that are introduced during DNA replication [7]. To better understand these mechanisms, it is crucial to explore factors that affect mutation rates.

The expression of DnaE2 during adverse conditions has been associated with increased mutation rates and drug resistance in mycobacteria [13]. However, due to the challenges of working with DnaE2 in isolation, biochemical studies involving DnaE2 have not yet been published. To work around these challenges, we employed a computational two-entropies analysis of homologous DnaE1 and DnaE2 sequences from mycobacteria to identify positions in the polymerases that could affect the polymerase fidelity. Next, we tested variants of DnaE1, harboring one or more mutations that make it more similar to DnaE2, in *M. smegmatis* through Cas9 knockdown of endogenous DnaE1 and rescue by the variant DnaE1. Variants with increased mutation rates were subsequently purified and analyzed *in vitro* for fidelity of DNA synthesis.

Polymerase fidelity is determined by two processes: nucleotide insertion fidelity and extension fidelity. The insertion fidelity selects the correct nucleotide opposite the template base. When the polymerase does incorporate an incorrect nucleotide, the distortion of the 3′ of the primer strand slows down or even prevents the incorporation of the next nucleotide, resulting in extension fidelity. Therefore, all high-fidelity DNA polymerases contain an exonuclease, as mis-incorporated nucleotides become roadblocks to DNA synthesis. As a result, inactivation of the exonuclease domain of replicative DNA polymerases leads to severe growth defects [44, 46, 47]. In contrast, low-fidelity Y family DNA polymerases such as the bacterial DNA Pol IV and Pol V, and the eukaryotic DNA polymerases Pol η, Pol κ, and Pol ι do not contain exonuclease domains, as their more open active site allows for both insertion and extension of mis-incorporated nucleotides. It was previously reported that DnaE2 lacks a residue in the PHP domain at the equivalent position of D228 in *M. smegmatis* DnaE1 (or D226 in *M. tuberculosis* DnaE1) that is required for exonuclease activity [16, 36, 44]. Therefore, given that a high-fidelity DNA polymerase without exonuclease activity leads to stalled DNA polymerase, it follows that DnaE2 must have a lower insertion and extension fidelity in order to function as a true error-prone DNA polymerase. However, due to the difficulty with working with purified DnaE2, this has not been validated. A pairwise comparison of DnaE1 and DnaE2 sequences shows only 25% sequence identity and 42% sequence similarity (Supplementary Fig. S3), making the identification of residues that may contribute to high-fidelity DNA synthesis impossible. Here we used a two-entropies analysis of 358 homologous DnaE1 and DnaE2 sequences from mycobacteria to identify 13 variants of DnaE1 that may contribute to DNA polymerase fidelity. Subsequent phenotypic and biochemical analysis revealed two residues in the palm domain, D431 and R432, that lower the fidelity of the polymerase when mutated to their corresponding DnaE2 residue, with no noticeable effect on cell growth. We furthermore show that DnaE1: D431S/R432D also incorporates more errors across different bases in a primer extension assay. These findings strongly suggest that the insertion fidelity of DnaE2 is lower than DnaE1, providing evidence that DnaE2 is an error-prone polymerase. Due to the high sequence identity of 86% between *M. tuberculosis* DnaE1 and *M. smegmatis* DnaE1, as well as the strong conservation observed in the DnaE2 alignment (Supplementary Fig. S3 and  S5), we believe that the functional insights gained from *M. smegmatis* will be highly relevant to *M. tuberculosis*. With DnaE2 being a key enzyme in the mycobacterial mutasome that has been linked to the rise of drug resistance in *M. tuberculosis*, our work provides new insights into the mechanism of drug resistance in the world’s deadliest infectious pathogen.

## Supplementary Material

gkaf1274_Supplemental_File

## Data Availability

The data underlying this article will be shared upon request to the corresponding author.

## References

[1] World Health Organization . Global Tuberculosis Report 2023, 2023.

[2] Mancuso G, Midiri A, Gaetano SD et al. Tackling drug-resistant tuberculosis: new challenges from the old pathogen *Mycobacterium tuberculosis*. Microorganisms. 2023;11:2277. 10.3390/microorganisms11092277. 37764122 PMC10537529

[3] Maitre T, Aubry A, Jarlier V et al. Multidrug and extensively drug-resistant tuberculosis. Médecine Et Maladies Infectieuses. 2017;47:3–10. 10.1016/j.medmal.2016.07.006. 27637852

[4] Dheda K, Mirzayev F, Cirillo DM et al. Multidrug-resistant tuberculosis. Nat Rev Dis Primers. 2024;10:1–27. 10.1038/s41572-024-00504-2. 38523140 PMC13335523

[5] Liebenberg D, Gordhan BG, Kana BD. Drug resistant tuberculosis: implications for transmission, diagnosis, and disease management. Front Cell Infect Microbiol. 2022;12:943545. 10.3389/fcimb.2022.943545. 36211964 PMC9538507

[6] Akalu TY, Clements ACA, Wolde HF et al. Economic burden of multidrug-resistant tuberculosis on patients and households: a global systematic review and meta-analysis. Sci Rep. 2023;13:22361. 10.1038/s41598-023-47094-9. 38102144 PMC10724290

[7] Dookie N, Rambaran S, Padayatchi N et al. Evolution of drug resistance in *Mycobacterium tuberculosis*: a review on the molecular determinants of resistance and implications for personalized care. J Antimicrob Chemother. 2018;73:1138–51. 10.1093/jac/dkx506. 29360989 PMC5909630

[8] Salini S, Bhat SG, Naz S et al. The error-prone polymerase DnaE2 mediates the evolution of antibiotic resistance in persister mycobacterial cells. Antimicrob Agents Chemother. 2022;66:e01773–21. 10.1128/aac.01773-21. 35156855 PMC8923199

[9] Pérez-Martínez DE, Zenteno-Cuevas R. SNPs in genes related to the repair of damage to DNA in clinical isolates of *M. tuberculosis*: a transversal and longitudinal approach. PLoS One. 2024;19:e0295464. 38917091 10.1371/journal.pone.0295464 PMC11198749

[10] Santos JA, Lamers MH. Novel antibiotics targeting bacterial replicative DNA polymerases. Antibiotics. 2020;9:776. 10.3390/antibiotics9110776. 33158178 PMC7694242

[11] Bosch B, DeJesus MA, Poulton NC et al. Genome-wide gene expression tuning reveals diverse vulnerabilities of *M. tuberculosis*. Cell. 2021;184:4579–92. 10.1016/j.cell.2021.06.033. 34297925 PMC8382161

[12] Ditse Z, Lamers MH, Warner DF. DNA replication in *Mycobacterium tuberculosis*. Microbiol Spectr. 2017;5. 10.1128/microbiolspec.TBTB2-0027-2016. PMC5507077 28361736

[13] Boshoff HIM, Reed MB, Barry CE et al. DnaE2 polymerase contributes to *in vivo* survival and the emergence of drug resistance in *Mycobacterium tuberculosis*. Cell. 2003;113:183–93. 10.1016/S0092-8674(03)00270-8. 12705867

[14] Warner DF, Ndwandwe DE, Abrahams GL et al. Essential roles for imuA’- and imuB-encoded accessory factors in DnaE2-dependent mutagenesis in *Mycobacterium tuberculosis*. Proc Natl Acad Sci USA. 2010;107:13093–8. 10.1073/pnas.1002614107. 20615954 PMC2919956

[15] Gessner S, Martin ZA-M, Reiche MA et al. Investigating the composition and recruitment of the mycobacterial ImuA′–ImuB–DnaE2 mutasome. eLife. 2023;12:e75628. 10.7554/eLife.75628. 37530405 PMC10421592

[16] Nasir N, Kisker C. Mechanistic insights into the enzymatic activity and inhibition of the replicative polymerase exonuclease domain from *Mycobacterium tuberculosis*. DNA Repair. 2019;74:17–25. 10.1016/j.dnarep.2018.12.006. 30641156

[17] Timinskas K, Balvočiūtė M, Timinskas A et al. Comprehensive analysis of DNA polymerase III α subunits and their homologs in bacterial genomes. Nucleic Acids Res. 2014;42:1393–413. 10.1093/nar/gkt900. 24106089 PMC3919608

[18] Oakley AJ . A structural view of bacterial DNA replication. Protein Sci. 2019;28:990–1004. 10.1002/pro.3615. 30945375 PMC6511741

[19] Sekurova ON, Sun Y-Q, Zehl M et al. Coupling of the engineered DNA “mutator” to a biosensor as a new paradigm for activation of silent biosynthetic gene clusters in Streptomyces. Nucleic Acids Res. 2021;49:8396–405. 10.1093/nar/gkab583. 34197612 PMC8373060

[20] Cai N, Chen J, Gao N et al. Engineering of the DNA replication and repair machinery to develop binary mutators for rapid genome evolution of Corynebacterium glutamicum. Nucleic Acids Res. 2023;51:8623–42. 10.1093/nar/gkad602. 37449409 PMC10484736

[21] Altschul SF, Gish W, Miller W et al. Basic local alignment search tool. J Mol Biol. 1990;215:403–10. 10.1016/S0022-2836(05)80360-2. 2231712

[22] Timinskas K, Venclovas Č. New insights into the structures and interactions of bacterial Y-family DNA polymerases. Nucleic Acids Res. 2019;47:4393–405. 10.1093/nar/gkz198. 30916324 PMC6511836

[23] Sievers F, Higgins DG. Clustal Omega for making accurate alignments of many protein sequences. Protein Sci. 2018;27:135–45. 10.1002/pro.3290. 28884485 PMC5734385

[24] Ye K, Lameijer E-WM, Beukers MW et al. A two-entropies analysis to identify functional positions in the transmembrane region of class A G protein-coupled receptors. Proteins. 2006;63:1018–30. 10.1002/prot.20899. 16532452

[25] Ye K, Vriend G, IJzerman AP. Tracing evolutionary pressure. Bioinformatics. 2008;24:908–15. 10.1093/bioinformatics/btn057. 18304936

[26] Sandberg M, Eriksson L, Jonsson J et al. New chemical descriptors relevant for the design of biologically active peptides. a multivariate characterization of 87 amino acids. J Med Chem. 1998;41:2481–91. 10.1021/jm9700575. 9651153

[27] Abagyan R, Totrov M. Biased probability Monte Carlo conformational searches and electrostatic calculations for peptides and proteins. J Mol Biol. 1994;235:983–1002. 10.1006/jmbi.1994.1052. 8289329

[28] Wong AI, Rock JM. CRISPR interference (CRISPRi) for Targeted Gene Silencing in Mycobacteria. Methods Mol Biol. 2021;2314:343–64. 34235662 10.1007/978-1-0716-1460-0_16

[29] Rock JM, Hopkins FF, Chavez A et al. Programmable transcriptional repression in mycobacteria using an orthogonal CRISPR interference platform. Nat Microbiol. 2017;2:1–9. 10.1038/nmicrobiol.2016.274. PMC5302332 28165460

[30] Castañeda-García A, Prieto AI, Rodríguez-Beltrán J et al. A non-canonical mismatch repair pathway in prokaryotes. Nat Commun. 2017;8:14246. 28128207 10.1038/ncomms14246 PMC5290159

[31] Arnold FM, Hohl M, Remm S et al. A uniform cloning platform for mycobacterial genetics and protein production. Sci Rep. 2018;8:9539. 10.1038/s41598-018-27687-5. 29934571 PMC6015033

[32] García-Nafría J, Watson JF, Greger IH. IVA cloning: a single-tube universal cloning system exploiting bacterial *in vivo* assembly. Sci Rep. 2016;6:27459. 27264908 10.1038/srep27459 PMC4893743

[33] Kuin RCM, Lamers MH, Westen GJPv. PyVADesign: a Python-based cloning tool for one-step generation of large mutant libraries. Bioinformatics. 2025;41:btaf433. 10.1093/bioinformatics/btaf433. 40888753 PMC12449254

[34] Green MR, Sambrook J. Molecular Cloning: A laboratory Manual, Vol. 1:4th edn. Cold Spring Harbor, N.Y: Cold Spring Harbour Laboratory Press, 2012.

[35] Nakata N, Kai M, Makino M. Mutation analysis of mycobacterial rpoB genes and rifampin resistance using recombinant *Mycobacterium smegmatis*. Antimicrob Agents Chemother. 2012;56:2008–13. 10.1128/AAC.05831-11. 22252831 PMC3318355

[36] Baños-Mateos S, van Roon A-MM, Lang UF et al. High-fidelity DNA replication in *Mycobacterium tuberculosis* relies on a trinuclear zinc center. Nat Commun. 2017;8:855. 10.1038/s41467-017-00886-w. 29021523 PMC5636811

[37] Maki H, Mo JY, Sekiguchi M. A strong mutator effect caused by an amino acid change in the alpha subunit of DNA polymerase III of *Escherichia coli*. J Biol Chem. 1991;266:5055–61. 10.1016/S0021-9258(19)67755-0. 2002048

[38] Gunasingam N . *Mycobacterium smegmatis*: exploring its similarities with *Mycobacterium tuberculosis*. Mycobacterial Diseases. 2023;13:1.

[39] Sparks IL, Derbyshire KM, Jacobs WR et al. *Mycobacterium smegmatis*: the vanguard of mycobacterial research. J Bacteriol. 2023;205:e00337–22. 36598232 10.1128/jb.00337-22 PMC9879119

[40] Sassetti CM, Boyd DH, Rubin EJ. Comprehensive identification of conditionally essential genes in mycobacteria. Proc Natl Acad Sci USA. 2001;98:12712–7. 10.1073/pnas.231275498. 11606763 PMC60119

[41] Floss HG, Yu T-W. Rifamycin—mode of action, resistance, and biosynthesis. Chem Rev. 2005;105:621–32. 10.1021/cr030112j. 15700959

[42] Ng WL, Rego EH. A nucleoid-associated protein is involved in the emergence of antibiotic resistance by promoting the frequent exchange of the replicative DNA polymerase in M. smegmatis. mSphere. 2024;9:e00122–24. 10.1128/msphere.00122-24. 38591887 PMC11237743

[43] Balint E, Unk I. For the better or for the worse? the effect of manganese on the activity of eukaryotic DNA polymerases. Int J Mol Sci. 2023;25:363. 10.3390/ijms25010363. 38203535 PMC10779026

[44] Rock JM, Lang UF, Chase MR et al. DNA replication fidelity in *Mycobacterium tuberculosis* is mediated by an ancestral prokaryotic proofreader. Nat Genet. 2015;47:677–81. 10.1038/ng.3269. 25894501 PMC4449270

[45] Jumper J, Evans R, Pritzel A et al. Highly accurate protein structure prediction with AlphaFold. Nature. 2021;596:583–9. 10.1038/s41586-021-03819-2. 34265844 PMC8371605

[46] Lancy ED, Lifsics MR, Kehres DG et al. Isolation and characterization of mutants with deletions in dnaQ, the gene for the editing subunit of DNA polymerase III in *Salmonella typhimurium*. J Bacteriol. 1989;171:5572–80. 10.1128/jb.171.10.5572-5580.1989. 2551891 PMC210399

[47] Slater SC, Lifsics MR, O’Donnell M et al. holE, the gene coding for the theta subunit of DNA polymerase III of *Escherichia coli*: characterization of a holE mutant and comparison with a dnaQ (epsilon-subunit) mutant. J Bacteriol. 1994;176:815–21. 10.1128/jb.176.3.815-821.1994. 8300534 PMC205119

