# Supplementary material for: Identification of determinants of high-fidelity DNA synthesis in Mycobacterium smegmatis DnaE1 through in silico and in vivo approaches
Source: Nucleic Acids Res. 2025 Nov 24;53(21):gkaf1274. doi: 10.1093/nar/gkaf1274 (PMC12641269; doi:10.1093/nar/gkaf1274)
Supplement: gkaf1274_Supplemental_File [file gkaf1274_supplemental_file.pdf]

SUPPLEMENTARY INFORMATION

Identification of determinants of high-fidelity DNA synthesis in *M. smegmatis* DnaE1 through *in silico* and *in vivo* approaches

R.C.M. Kuin[1,2], G.J.P. van Westen[1], M.H. Lamers[2]

[1] Medicinal chemistry, Leiden University, Einsteinweg 55, 2333 CC Leiden, the Netherlands
[2] Department of Cell and Chemical Biology, Leiden University Medical Center, Albinusdreef 2 2333 ZA Leiden, the Netherlands

Supplementary Table 1. DNA substrates used for primer extension assay.

**Oligo**	**Sequence (5′ – 3′ direction)**	
	**Manganese dependent assay**	**Time-course assay**
**TempAmm**	ggtcgcgtcgAgctctgtGGACGAAGGACTCCCAAC	ggtcgcgtcgAgctctgtGGACGAAGGACTCCCAAG
**TempCmm**	ggtgaggtagCgatgagtGGACGAAGGACTCCCAAC	*same*
**TempGmm**	cctcacctacGcatcactGGACGAAGGACTCCCAAC	cctcacctacGcatcactGGACGAAGGACTCCCAAG
**TempTmm**	ggacgcgacgTgcacagaGGACGAAGGACTCCCAAC	*same*
**Primer**	GTTGGGAGTCCTTCGTCC	6-FAM-CTTGGGAGTCCTTCGTCC

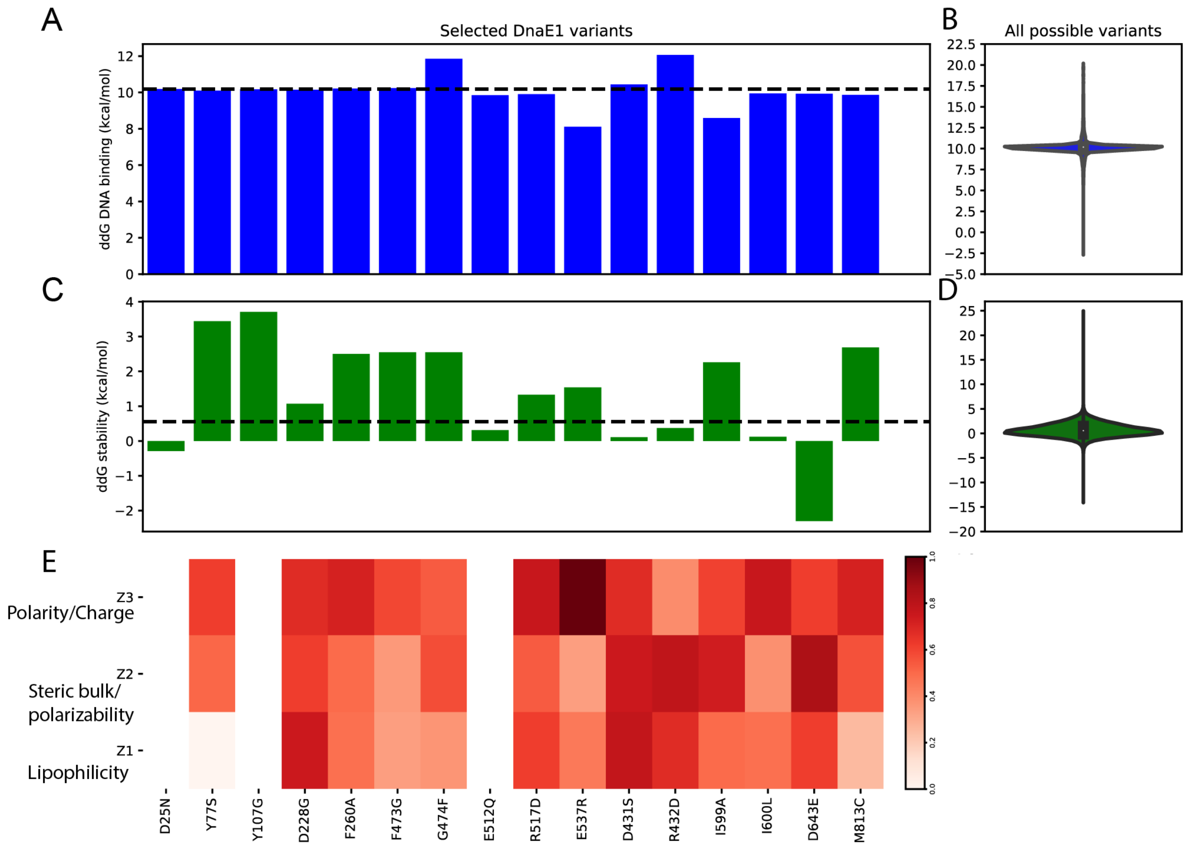

Supplementary Figure S1. Properties of DnaE1 variants, based on a two-entropies analysis. (A) Predicted change in binding free energy (ddG) for DNA binding (kcal/mol) upon mutation of a single residue for selected variants and **(B)** all possible DnaE1 variants. **(C)** Predicted effect on DnaE1 stability (ddG) upon mutation for selected variants and **(D)** all possible variants. Calculations were done in ICM-Pro version 3.9-3a (Molsoft L.L.C.) and the black dotted line represents the median value based on all possible variants. **(E)** Heatmap highlighting the differences in physicochemical properties for selected positions in the Multiple Sequence Alignment. No data is shown for control variants D25N, Y107G and for E512Q. Lipophilicity, steric properties (Steric bulk/Polarizability) and electronic properties (Polarity/Charge) are represented by Z1, Z2 and Z3 respectively (26). Scaled standard deviations of these physicochemical properties are shown, where a higher standard deviation indicates a bigger difference in the property for all residues in that position of the Multiple Sequence Alignment.

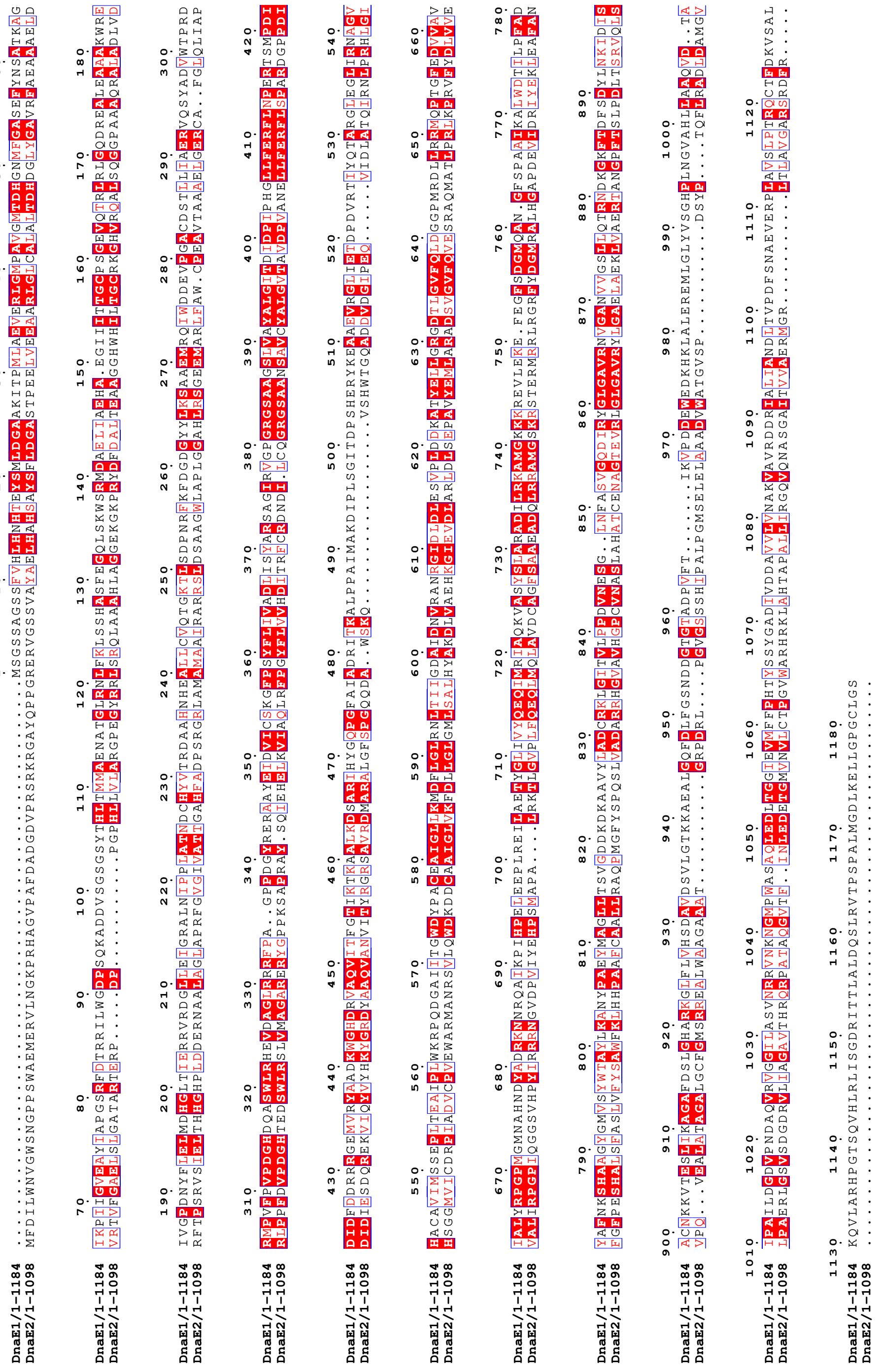

Supplementary Figure S2. Alignment of *M. tuberculosis* DnaE1 and DnaE2 protein sequences. Identical residues are marked in red background and white letters, similar residues are marked with red letter in blue box. Overall sequence identity = 25%, overall sequence similarity = 45%. Figure was created with ESPript version 3.0 (1).

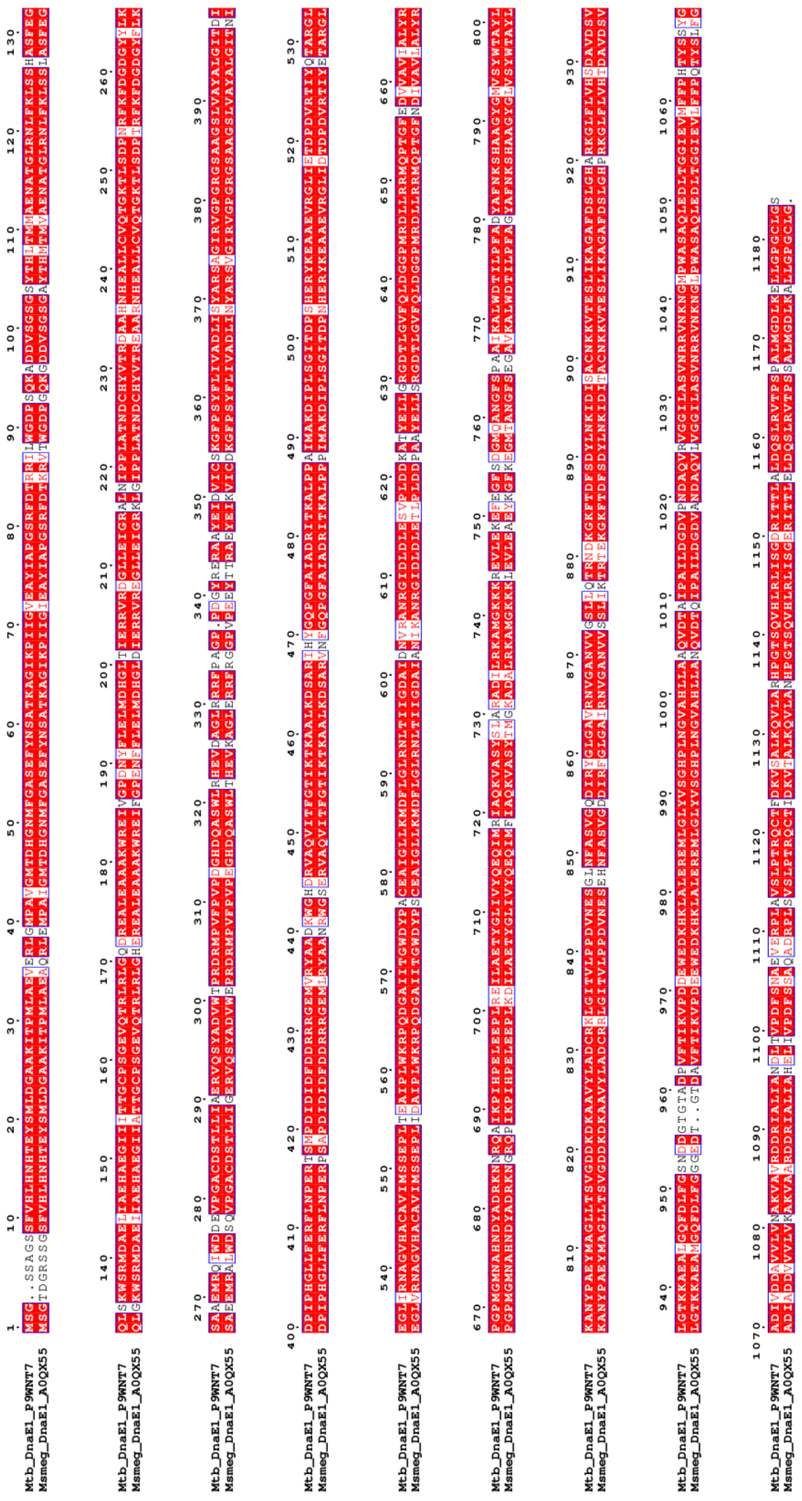

Supplementary Figure S3. Alignment of *M. tuberculosis* DnaE1 and *M. smegmatis* DnaE1 protein sequences. Identical residues are marked in red background and white letters, similar residues are marked with red letter in blue box. Overall sequence identity = 86%, overall sequence similarity = 96%. Figure was created with ESPript version 3.0 (1).

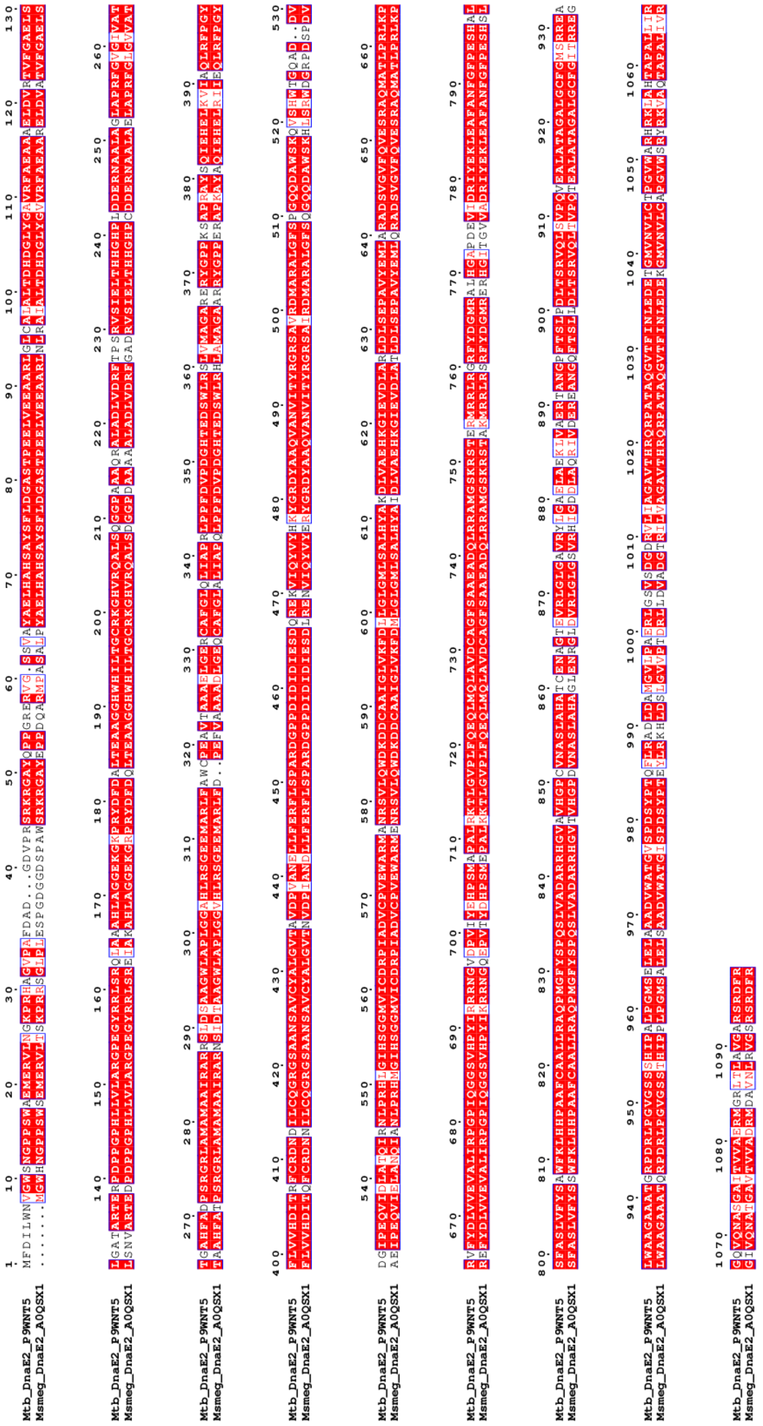

Supplementary Figure S4. Alignment of *M. tuberculosis* DnaE2 and *M. smegmatis* DnaE2 protein sequences. Identical residues are marked in red background and white letters, similar residues are marked with red letter in blue box. Overall sequence identity = 82%, overall sequence similarity = 94%. Figure was created with ESPript version 3.0 (1).

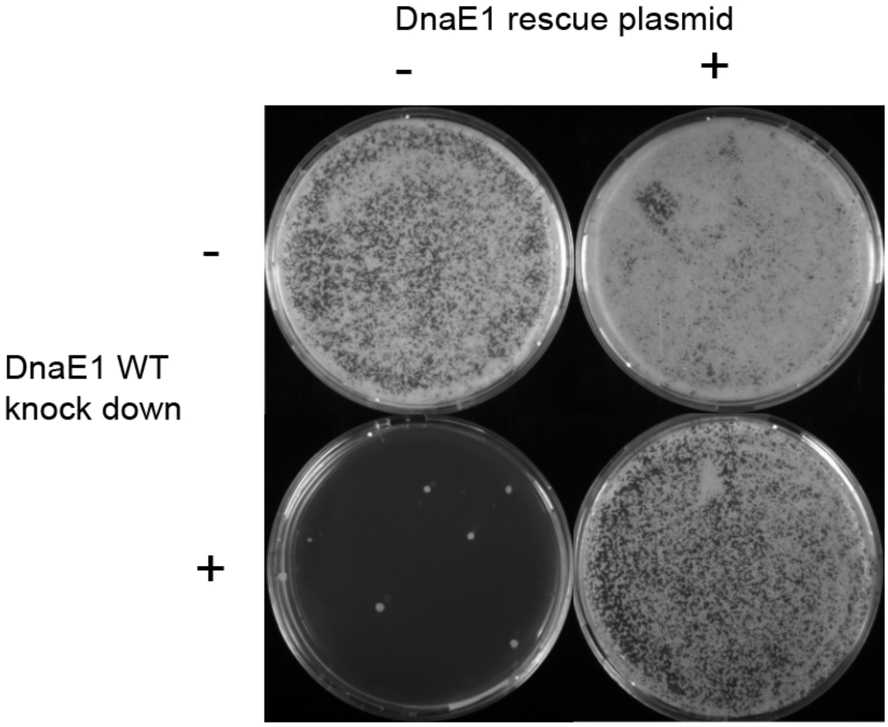

Supplementary Figure S5. dCas9 Knock-down and rescue of DnaE1. *M. smegmatis* cells containing dCas9 plasmid with sgRNA targeting the endogenous DnaE1 gene and a dCas9-resistant expression plasmid of DnaE1 gene in absence and presence of dCas9 and rescue DnaE1.

Supplementary Table 2. PHP variants of *M. smegmatis* DnaE1 used in this study that do not show enhanced mutation frequencies.

			cell growth		Mutation frequency	
	Mtb residue	Domain	- WT DnaE1	+ WT DnaE1	- WT DnaE1	+ WT DnaE1
**Y77S**	75	PHP	∅	<	n.d.	0.6 ± 0.3
**D80-106**	78-104	PHP	∅	<	n.d.	0.6 ± 0.3
**D228G**	226	PHP	∅	<	n.d.	0.5 ± 0.3

Effect on cell growth upon knockdown of endogenous dnaE1 and rescue by variants, measured across three biological replicates. ∅ indicates that there was no growth, < indicates that there was less growth compared to the WT and = indicates that there was equal growth as compared to the WT, four days after transformation. Effect of the DnaE1 variant on mutation frequencies, determined using a rifampicin resistance assay, reported as fold increase (n.d. = not determined) ± the standard deviation across three biological replicates.

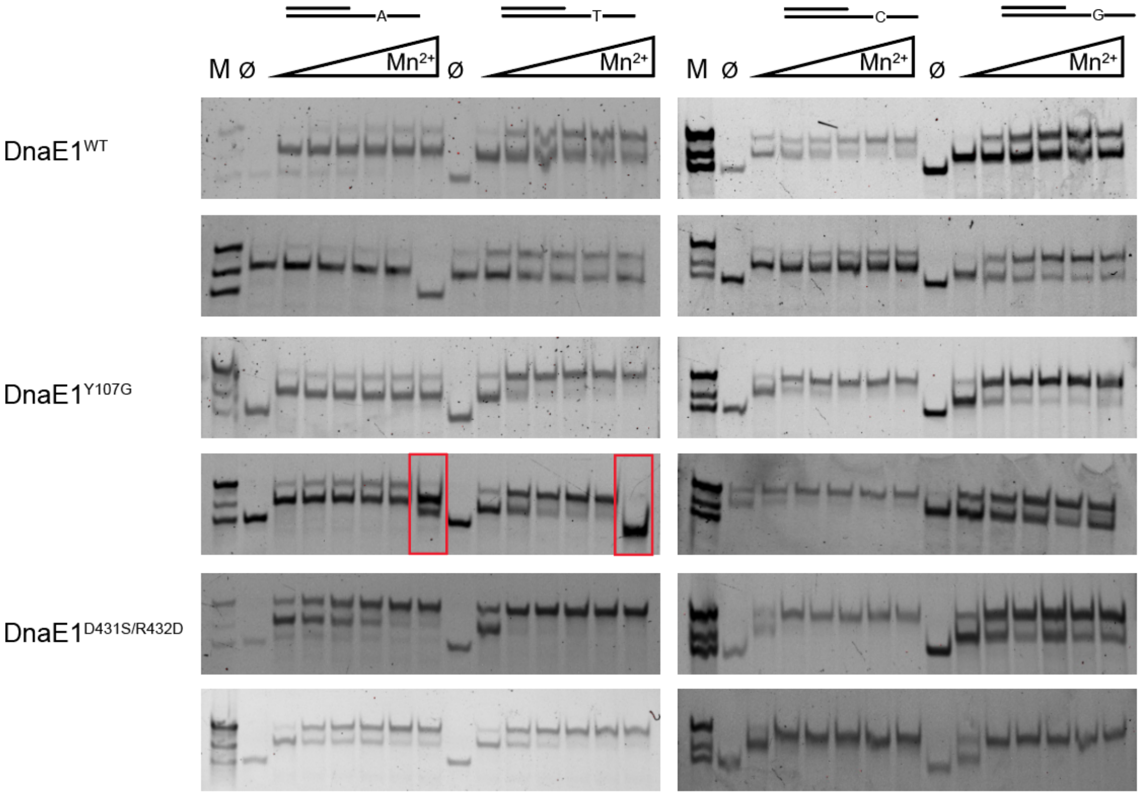

Supplementary Figure S6. Gels used for the densitometric analysis shown in Figure 3B. Lanes outlined in red were excluded because they were performed with 10 mM Mn^{2+} instead of 5 mM.

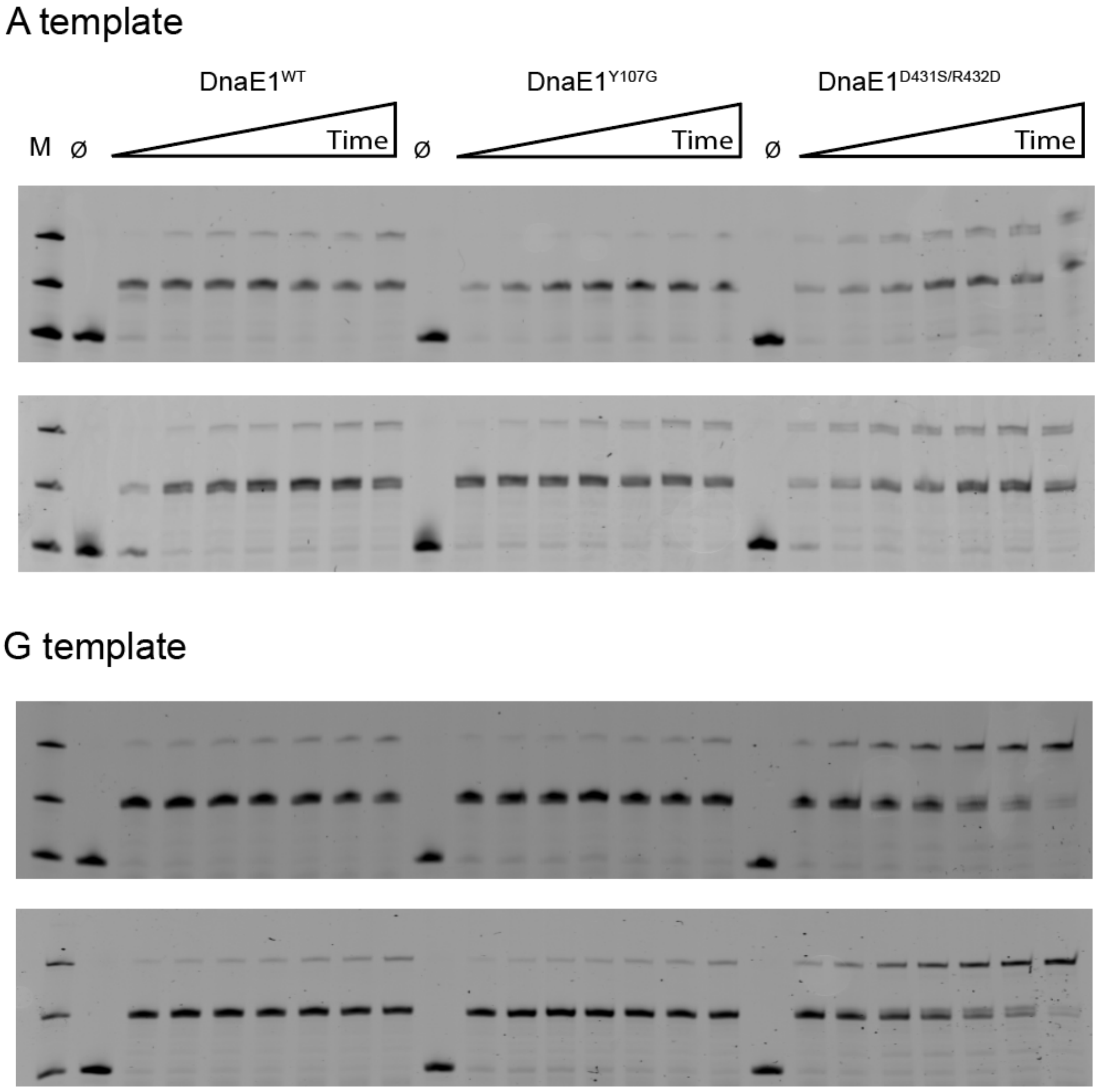

Supplementary Figure S7. Additional gels used for the densitometric analysis shown in Fig. 3D.

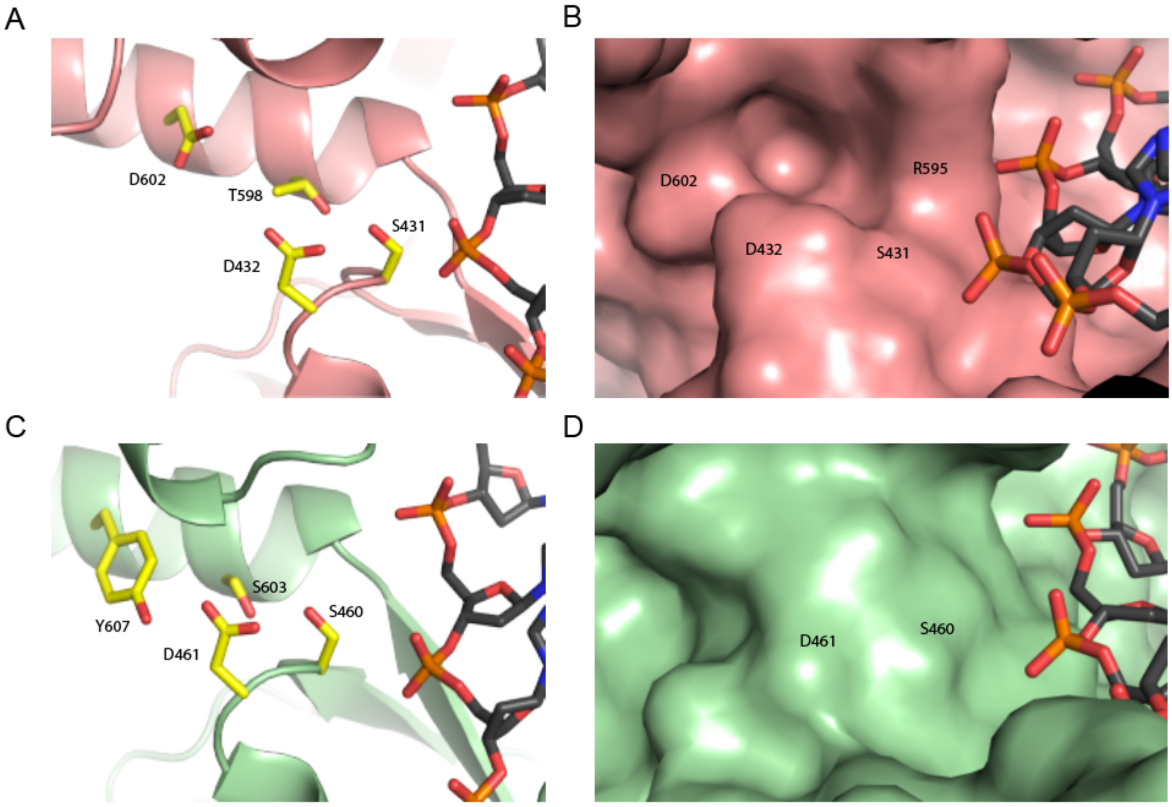

Supplementary Figure S8. (A) Location of the S431 and D432 in the palm domain of *M. smegmatis* DnaE1, shown in an AlphaFold model of the D431S/R432D variant. (B) Surface representation of the palm domain of DnaE1, highlighting a groove that could accommodate the DNA template strand. (C) AlphaFold model of DnaE2. (D) Surface representation of the palm domain of DnaE2, showing that the groove observed in DnaE1 is absent.

Supplementary References

1. Robert,X. and Gouet,P. (2014) Deciphering key features in protein structures with the new ENDscript server. *Nucleic Acids Research*, 42, W320–W324.
